# Screening Currency Notes for Microbial Pathogens and Antibiotic Resistance Genes Using a Shotgun Metagenomic Approach

**DOI:** 10.1371/journal.pone.0128711

**Published:** 2015-06-02

**Authors:** Saakshi Jalali, Samantha Kohli, Chitra Latka, Sugandha Bhatia, Shamsudheen Karuthedath Vellarikal, Sridhar Sivasubbu, Vinod Scaria, Srinivasan Ramachandran

**Affiliations:** 1 GN Ramachandran Knowledge Center for Genome Informatics, CSIR Institute of Genomics and Integrative Biology (CSIR-IGIB), Mathura Road, Delhi, 110 020, India; 2 Functional Genomics Unit, CSIR Institute of Genomics and Integrative Biology (CSIR-IGIB), New Delhi, India; 3 Structural Biology Unit, CSIR—Institute of Genomics and Integrative Biology, Mathura Road, New Delhi, 110 020, India; 4 Respiratory Disease Biology Unit, CSIR- Institute of Genomics and Integrative Biology, Mall Road, Delhi, 110007, India; 5 Genomics and Molecular Medicine Unit, CSIR Institute of Genomics and Integrative Biology, Mathura Road, Delhi, India; 6 Academy of Scientific and Innovative Research (AcSIR), CSIR IGIB South Campus, Mathura Road, Delhi, 110020, India; Cairo University, EGYPT

## Abstract

Fomites are a well-known source of microbial infections and previous studies have provided insights into the sojourning microbiome of fomites from various sources. Paper currency notes are one of the most commonly exchanged objects and its potential to transmit pathogenic organisms has been well recognized. Approaches to identify the microbiome associated with paper currency notes have been largely limited to culture dependent approaches. Subsequent studies portrayed the use of 16S ribosomal RNA based approaches which provided insights into the taxonomical distribution of the microbiome. However, recent techniques including shotgun sequencing provides resolution at gene level and enable estimation of their copy numbers in the metagenome. We investigated the microbiome of Indian paper currency notes using a shotgun metagenome sequencing approach. Metagenomic DNA isolated from samples of frequently circulated denominations of Indian currency notes were sequenced using Illumina Hiseq sequencer. Analysis of the data revealed presence of species belonging to both eukaryotic and prokaryotic genera. The taxonomic distribution at kingdom level revealed contigs mapping to eukaryota (70%), bacteria (9%), viruses and archae (~1%). We identified 78 pathogens including *Staphylococcus aureus*, *Corynebacterium glutamicum*, *Enterococcus faecalis*, and 75 cellulose degrading organisms including *Acidothermus cellulolyticus*, *Cellulomonas flavigena* and *Ruminococcus albus*. Additionally, 78 antibiotic resistance genes were identified and 18 of these were found in all the samples. Furthermore, six out of 78 pathogens harbored at least one of the 18 common antibiotic resistance genes. To the best of our knowledge, this is the first report of shotgun metagenome sequence dataset of paper currency notes, which can be useful for future applications including as bio-surveillance of exchangeable fomites for infectious agents.

## Introduction

Paper currency notes are extensively used in barter and trade [1]. There is well documented evidence suggesting that currency notes could act as fomites with enormous potential to carry microbes [2]. Paper currency has rough surface that provides a good niche for microorganisms and other particulates to settle and accumulate over long term and thereby constitute a potential source of infection. The levels and diversity of microbial contamination of currency notes depend on several factors including the period of their circulation, handling and its texture. The capacity of currency notes to absorb moisture also facilitates the growth and viability of microorganisms. Recently, several attempts have been made to characterize the sojourning microbial population on currency notes in various parts of the world [3–9].

Currency notes contaminated with pathogenic bacteria can also be a potential source of infection as in the case of food poisoning caused by enteropathogens [10,11]. In addition, currency notes have also been evaluated for their potential to transmit infectious pathogens like *Ascaris lumbricoides*, *Enterobius vermicularis*, *Trichuris trichiura* and *Taenia species* [1]. Carrier micro-organisms apart from reducing the lifespan of the notes, have been documented to cause infections in the skin, eye, gastrointestinal tract [8,12], internal organs [13,14], as well as the respiratory tract [15] in humans. Microorganisms such as *Micrococcus* spp., *Corynebacterium* spp., *Vibrio cholerae*, *Mycobacterium tuberculosis* and members of the *Enterobacteriacea* family top the list subsequently. As early as 1972, Abrams and Waterman reported pathogenic microbes such as *Staphylococcus aureus*, *Escherichia coli*, *Klebseilla* spp. and Enterobacter spp. on the banknotes from The United States of America (USA) [9]. Recently, Elumalai et al. isolated eight different types of bacterial species *E*. *coli*, *Proteus mirabilis*, *Vibrio* spp., *S*. *aureus*, *Pseuodomonas* spp., *Salmonella* spp., *Bacillus* spp., *and Klebsiella* spp. from 30 Indian currency notes consisting of five notes each of Indian Rupee ₹ 5 and ₹ 10 denominations [16]. These species are well known to cause a wide variety of diseases ranging from food poisoning, wound and skin infections, respiratory and gastrointestinal problems to life threatening diseases such as meningitis and septicemia [16]. In the interest of the public health, Australia withdrew all its circulating paper currency notes and replaced them with plastic currency [17].

Several studies have reported high levels of bacterial contamination in commonly circulated currency notes [7,8,18–21]. The techniques used have been culture-dependent, which severely restricts the identification and characterization of un-culturable microbes [22,23]. The members of the bacterial families *Bacillus* spp., and *Staphylococcus* spp. are the most common contaminants isolated from paper currency. A recent study had also highlighted the potential of currency notes to harbor drug-resistant pathogens [24]. Metagenome sequencing is a new widely used approach which has been made more feasible by high-throughput sequencing techniques and also has the potential to identify microbial population in a given sample without employing tedious microbial culture and isolation. In this respect this technology offers unique opportunity to study microbes, which are either difficult or not culturable in laboratory conditions [25,26]. A variety of complementary techniques have been used to investigate metagenomes. While the time honored 16S rRNA sequencing approaches provide valuable insights into taxonomic identities of the organisms in the microbiome, recent shotgun sequencing techniques offer additional insights into the gene repertoire. In addition, the gene repertoire could be further used to examine and characterize the biological pathways [27,28], and permit discovery of novel metabolic enzymes with potential to catalyze reactions forming new metabolites [29–31]. In addition, the available public sources for gene function annotation and compendia of specific activities including annotations of antibiotic resistance genes offer new opportunities to combine shotgun metagenomics sequence data and to probe the annotation of microbial diversity and their genes in the environmental niches. [32–34]

In the present study, we have used a shotgun metagenome sequence approach to investigate the microbial diversity including the pathogens present on Indian currency notes. Further, we screened the gene repertoire for identifying potential antibiotic resistance genes. The gene repertoire was also further classified to evaluate the various biological pathways active in the metagenome. Our analysis suggests a significant diversity in the microbial population on paper currency notes and presence of antibiotic resistance genes. The current study presents the most comprehensive metagenomic analysis of currency notes and provides interesting insights into the microbial diversity and the drug resistance patterns of several pathogens and other present micro-organisms.

## Material and Methods

### Sample collection

Sample sets were selected on the basis of the Reserve Bank of India statistics of circulation of Indian currency for the year 2011–2012 (http://www.rbi.org.in/scripts/AnnualReportPublications). We selected the most and the least circulated ₹ denominations, ₹ 10 (32.6%), ₹ 100 (20.75%) and ₹ 20 (4.8%), based on circulation proportions. On being released by the apex bank, the currency notes are exclusively circulated across the country. Samples were collected in sterile plastic bags from random spots such as street vendors, grocery shops, snack bars, canteen, tea shops, hardware shops, chemists, etc. across the Delhi city metropolitan area (Geographic coordinates 28.6100° N, 77.2300° E), which is home to 1.67 million individuals and approximately 1.38 percent of the Indian population [35]. We collected three sets of currency notes, originating in the same year 2011–12: ₹ 10 currency notes (C1); ₹ 100 currency notes (C2) and ₹ 20 currency notes (C3). An empirical set of four currency notes were pooled from the collection and samples were processed in duplicates. In order to independently verify the results of antibiotic resistance genes from the first phase of the study, we collected additional three sets of currency notes, in duplicates, originating in the year 2011–12: ₹ 10 currency notes (C1a and C1b); ₹ 100 currency notes (C2a and C2b) and ₹ 20 currency notes (C3a and C3b).

### Metagenomic DNA isolation

Four currency notes of each sample (in duplicates) were placed in autoclaved boxes and suspended in 15 ml of DNA extraction buffer (DEB) (100 mM Tris-HCl (pH 8), 100 mM EDTA (pH 8.0), 1.5M NaCl, 100mM sodium phosphate (pH 8.0), 1% cetyl trimethyl ammonium bromide (CTAB) (Sigma, USA) and 40 μl Proteinase K (Sigma, USA) (10 mg/ml). The boxes were incubated at 37°C for 10–12 hours under constant shaking at 180 rpm. The samples were re-extracted with 1 ml of DEB and the entire extraction buffer was transferred into 50 ml tubes. Supernatant containing the metagenomic samples was collected by low speed centrifugation at 5000 rpm for 10 min at room temperature. To the supernatant, 1 ml of sodium dodecyl sulfate (20% w/v) (Sigma, USA) and 20 μl of Proteinase K (10 mg/ml) (Sigma, USA) were added and incubated at 65°C for two hours. The solution was centrifuged at 10,000 rpm for 15 min at 4°C. The upper aqueous phase was extracted with equal volume of chloroform: isoamylalcohol (24:1) by centrifugation at 10,000 rpm for 20 min at room temperature. DNA was subsequently precipitated by adding two volumes of chilled 70% ethanol. DNA precipitates were collected by centrifugation at 10,000 rpm for 10 min at 4°C, air dried and re-suspended in 20 μL sterile Tris-EDTA buffer. The duplicates were pooled into single tubes for further use, except in the case of samples used in validation study. Extracted DNA samples were stored at −20°C.

### DNA quality and quantity check

Extracted DNA was quantified using the Infinite 200 PRO Nano Quant microplate reader (Tecan Technologies Inc). Absorbance ratio at 260nm/280nm (DNA/protein) was determined to evaluate the purity. The DNA concentration was estimated using picogreen assay (Invitrogen Quant-iT PicoGreen). The integrity of the DNA samples were assessed by electrophoresing 2 μl of each sample on a 0.8% agarose gels in 1 X Tris-Acetate-EDTA buffer. Gels were stained with ethidium bromide and viewed using Gel imaging system (Syngene Technologies Inc).

### Library preparation and sequencing

Approximately 2 μg of quality checked metagenomic DNA from each sample was used for library construction and sequencing. Total DNA was fragmented using Covaris S220 system to obtain an average insert size of approximately 200 bp. The ends were repaired by ligation with specific index adapters. The fragmented DNA was electrophoresed on a 2% agarose gel and fragments of an approximate length of 400 bp were eluted out. Further amplification of the selected DNA fragments were carried out in order to obtain the library. The library was quantified and pooled using equimolar concentrations to perform multiplex sequencing. Clusters were generated on the flow cell using cBot Paired end cluster generation kit (Illumina Inc., USA) as per manufacturer’s instructions. The libraries were sequenced by 200 bp chemistry from both ends for 101 cycles using Illumina Hiseq 2500.

### Genome assembly and analysis

#### Read quality assessment

**Fig 1:**
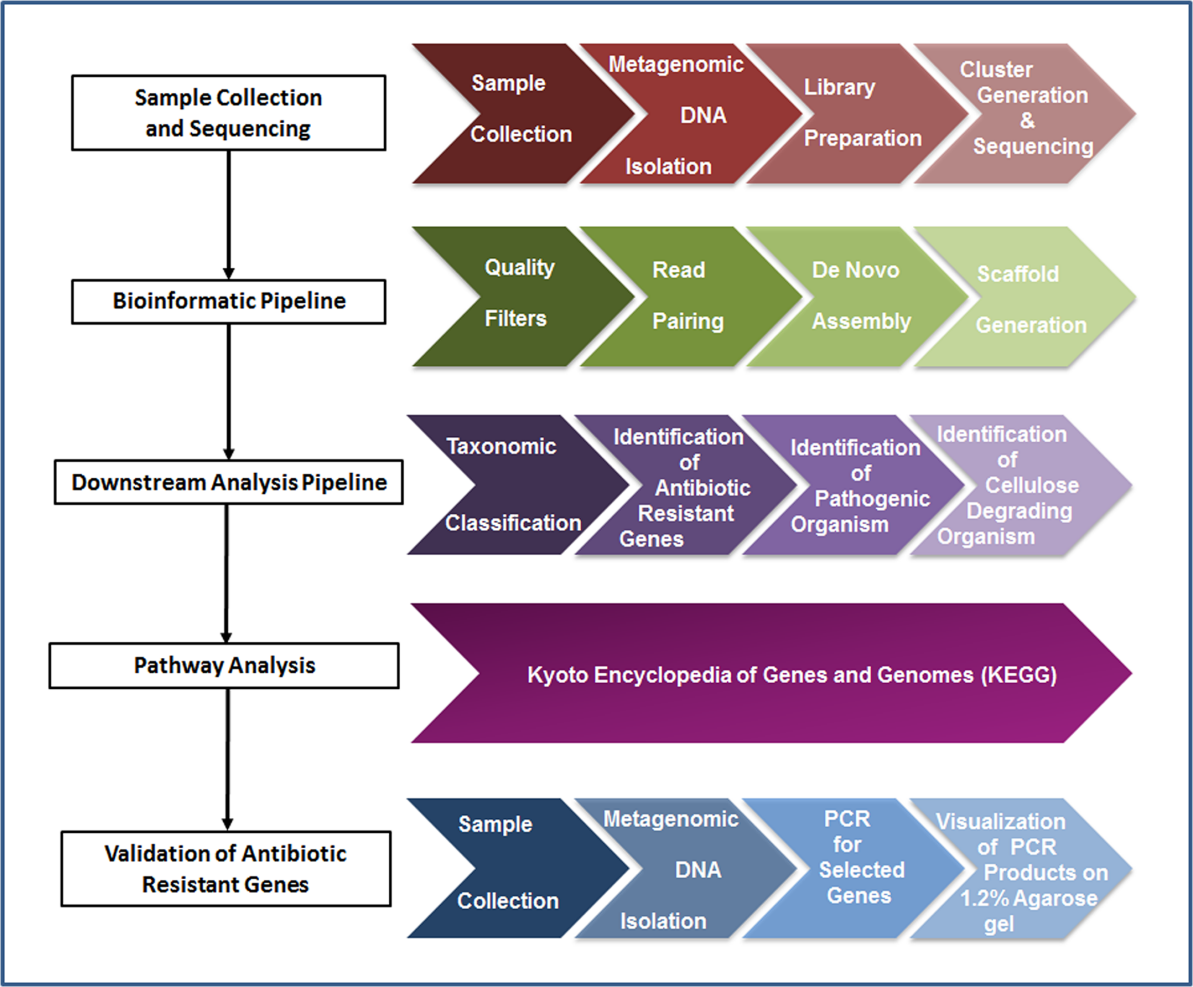
The complete workflow for DNA sequence quality check and sequence analysis.

The paired end reads in FASTQ format generated for the three samples C1, C2 and C3 from the Next Generation Sequencing (NGS) platform were examined for read length, total number of reads, percentage GC and mean base quality distribution using the FastQC toolkit [36]. FastX toolkit (http://hannonlab.cshl.edu/fastx_toolkit) [37] was used to calculate the sequence duplication rate. The reads were filtered out using a cutoff value of Phred score > 30 and length sorted at ≥ 70 bases using Perl scripts included in SolexaQA [38,39]. Ambiguous bases (including N) were not included in any downstream analysis. The complete workflow for DNA sequence quality check and sequence analysis is shown in Fig 1.

#### *De novo* assembly

The trimmed reads for respective samples were assembled *de novo* using MetaVelvet-v1.2.01, a De-Brujin graph based *de novo* assembler [40]. *De novo* assembly was attempted on each dataset, with different k-mers ranging from 31 to 63. The individual k-mer files for each sample were concatenated into a single file. These concatenated files for each sample namely, C1, C2 and C3 were assembled into larger contigs with minimum contig length of 100 bases using CLC Genomics Workbench 6.5.1 software (CLC bio) [41].

#### Taxonomic identification

The contigs were binned into taxonomic groups by the Lowest Common Ancestor (LCA)-based algorithm implemented in MEGAN software (version 4.70.4) [42]. The publicly available NCBI-NT database (http://www.ncbi.nlm.nih.gov/) was used to identify the taxonomic groups up to the species level in the metagenomic contigs. The parameters used to assign the taxonomic identity to a metagenomic contig included a min support, min score and top percent. Min support is minimum number of reads required for a taxon to appear in the result, which was set to the default value of 5. Min score is the lowest threshold for the bit score of hits, which was set to the default value of 50. Top percentage is a threshold for the maximum percentage by which the score of a hit may fall below the best score for a given read, which was set to the default value of 10%. MEGAN compares the input contigs with the NCBI-NT database and assigns taxonomy to each contig to generate a dendrogram according to the above set parameters. After taxonomic dendrogram generation, MEGAN was further used to estimate the relative abundance of the identified Operational Taxonomic Units (OTUs) from different samples.

#### Comparative analysis

Comparative analysis was carried out by mapping individual sample reads onto scaffolds for coverage calculations. To evaluate the coverage for each contig, the paired end reads for each sample were mapped independently to all the individual assemblies using Bowtie 2.0.0-beta7 software [43]. We used a normalized measure of the contig coverage Reads mapped Per Kilobase contig per Million reads (RPKM) for assessing the relative copy number representing the individual assembled contigs. This measure has been extensively used to estimate copy numbers for nucleotides especially in RNA sequencing experiments [44].

#### Pathway analysis

The set of contigs from all three datasets was analyzed for the presence of various pathways, using Kyoto Encyclopedia of Genes and Genomes (KEGG) [45] in terms of the enzyme annotation for the predicted genes encoded by the contigs. Comparison of the genes and pathways in the contigs set was performed using bespoke scripts. Additionally, enzymes present in all three sets and enriched exclusively in one set were identified. The pathways representation was generated using KEGG Mapper [46].

#### Pathogenic and cellulose degrading organisms

The combined set of contigs from all three datasets was scanned to identify the pathogenic and cellulose degrading organisms present on Indian currency notes using the publicly available databases [47–51] and matched with the species list identified by MEGAN for each contig.

#### Annotation of antibiotic resistance genes

Characterization and annotation of the antibiotic resistance genes from all three sets of samples were carried out using the Antibiotic Resistance Genes Database (ARDB) [52]. The database consisted of 13,293 antibiotic resistance genes classified into 377 types based on their resistance profiles and sequence similarity. These genes were found to be implicated in resistance to 257 antibiotics. The ARDB annotation tool (ardbAnno.pl) uses the ARDB datasets to identify and annotate potential antibiotic resistance genes using BLAST similarity search.

#### PCR validation for antibiotic resistance genes

We examined for the presence of two antibiotic resistance genes i.e. *aph3ia* (or *aphA1)* and *baca* in the samples C1a, C1b, C2a, C2b, C3a and C3b collected separately and independently. The two primer sets used are listed in Table 1 [53]. The specificities of oligonucleotide sequences for the target organisms were also checked with BLAST (online tool). The primers were synthesized commercially by Integrated DNA Technologies, Inc. (USA). Each PCR mixture (20 μl) contained 2 μM concentrations of the deoxynucleoside triphosphates, 0.5 μM primer, 50 ng of template metagenomic DNA, 1.5 U of Taq DNA polymerase (GeNei) in Taq Buffer A (GeNei) containing 1.5 mM MgCl_2_. The optimal annealing temperature for each primer set was determined using Mastercycler gradient PCR machine (PTC- 200 Peltier Thermal Cycler, Atlantic Lab Equipment, Salem, MA). Table 1 enlists the PCR program and PCR product size for both the genes.

**Table 1:** PCR primer sequences and cycling conditions for antibiotic resistance genes.

Antibiotic resistance gene	Primer Sequences	PCR cycling conditions	Product Size (bp)
*aph3ia*	FP: 5’ TGAACAAGTCTGGAAAGAAATGCA 3’	***I*** 94°C 5’; ***D*** 94°C 1’, ***A*** 51.4°C 30”, ***E*** 72°C 30”; 35 ***cy***; ***FE*** 72°C 10’	113bp
	RP: 5’ CCTATTAATTTCCCCTCGTCAAAAA 3’		
*baca*	FP: 5’ TTCCACGACACGATTAAGTCATTG 3’	***I*** 94°C 5’; ***D*** 94°C 1’, ***A*** 53.9°C 30”, ***E*** 72°C 30”, 35 ***cy***; ***FE*** 72°C 10’	107bp
	RP: 5’ CGGCTCTTTCGGCTTCAG 3’		

FP = forward primer; RP = reverse primer; I = initial denaturation; D = denaturation; A = annealing; E = extension; cy = cycles; FE = final extension.

## Results

### Metagenomic DNA sequencing

The DNA concentration of all the samples ranged between 350–850 ng/μl and the absorbance ratio was approximately 1.8. We generated a total of 2.2 Gigabases (Gb), 3.29 Gb and 4.06 Gb for the sample sets C1, C2 and C3 respectively. Each of the reads was of 101 bases, in a paired-end format, with an average insert size of 250 bases. The average GC content for all the reads was 41%. The average base Phred quality score for the reads ranged from 30 to 39 upto 70 bases; thereafter the quality dropped below 30. The reads were trimmed to a length less than 70 bases covering upto Phred score >30 and were considered for assembly. Salient features of the read statistics are summarized in Table 2.

**Table 2:** Sequencing reads and contigs statistics for each of the three datasets.

Sample ID	Raw reads	Filtered reads	Reads mapping on human RefSeq (%)	N50	Minimum length (bp)	Maximum length (bp)	Total number of contigs
Currency 1 (C1)	4,502,445	3,464,046	5,26,535 (15.20)	223	100	23,289	3,53,751
Currency 2 (C2)	6,737,172	5,421,442	8,61,467 (15.89)	162	100	9,557	5,51,759
Currency 3 (C3)	8,319,810	5,982,243	3,31,416 (25.54)	154	100	33,939	9,19,333

### Metagenome assembly

The reads were initially mapped to the Human genome (Feb 2009, GRCh37/hg19). Among the three samples, the mapping proportions to the reference human genome were 15.20%, 15.89% and 25.54% respectively. The detailed contig statistics are shown in Table 2. The *de novo* assembly of the metagenome had a significantly larger contig size, with an average N50 of over 180 bases.

### Taxonomic identification

**Fig 2:**
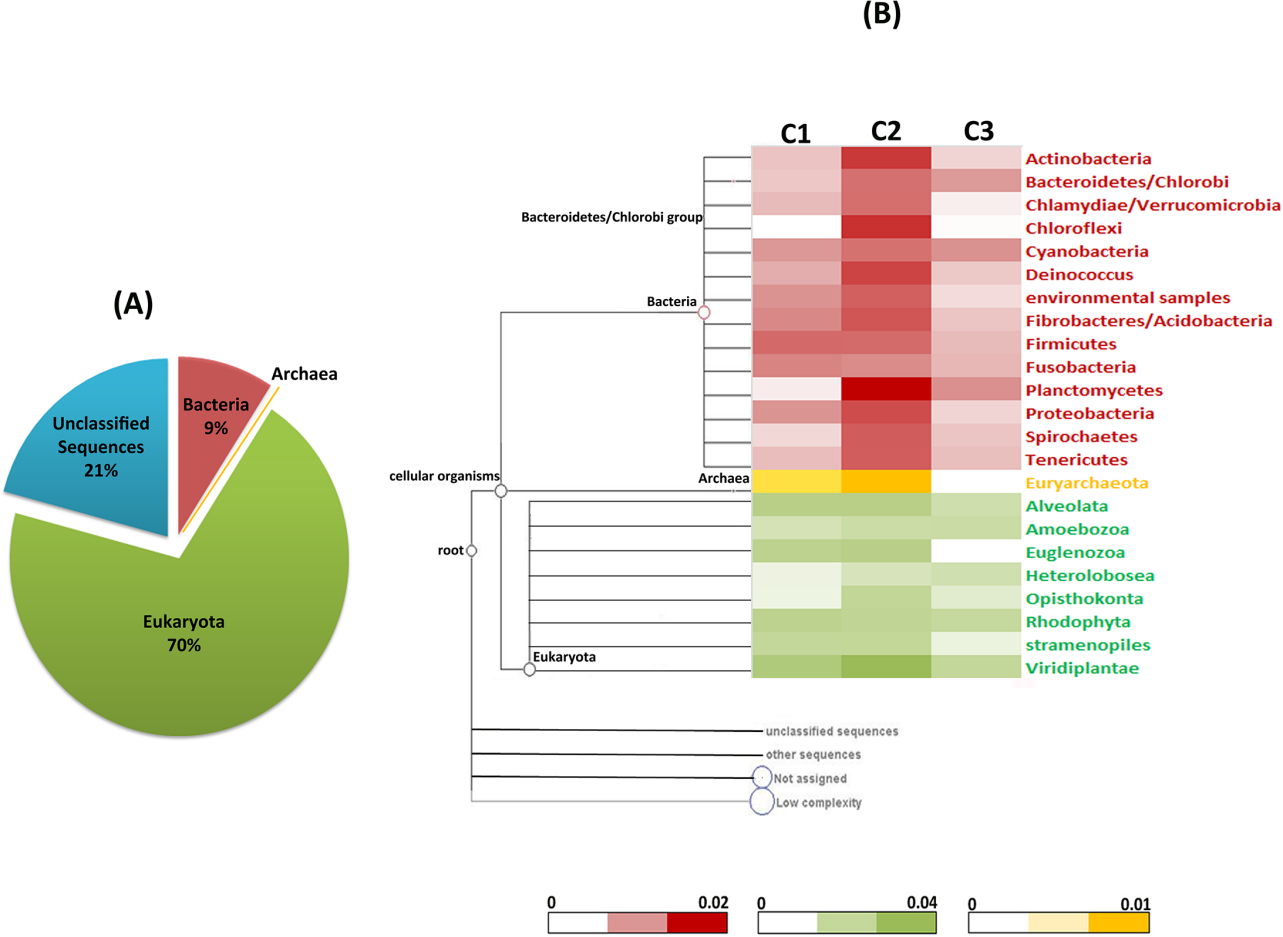
Taxonomic distribution of the species in the metagenome of Indian currency notes using MEGAN & relative microbial load for all the three samples. **(A)** The overall proportion of each Kingdom found in all the three samples. **(B)** Heatmap representation of the relative microbial load in the range shown in sale bars on the three currency samples C1, C2, C3. The red, green and yellow color represents bacteria, eukaryota and archaea phyla respectively.

As represented in Fig 2A, the overall taxonomic distribution at kingdom level for all the samples revealed that the highest proportion of contigs mapped to eukaryota (70%), followed by bacteria (9%), viruses and archae (~1%). The overall taxonomic distribution for all the samples revealed eight phyla of eukaryote, 14 phyla of bacteria and one phylum of archaea (Fig 2B). It was observed that the contigs mapped to eukaryota were proportionally distributed in all the three samples; however, samples C3 and C1 had maximum contig coverage for bacteria and viruses, respectively.

### Comparative analysis of three samples

**Fig 3:**
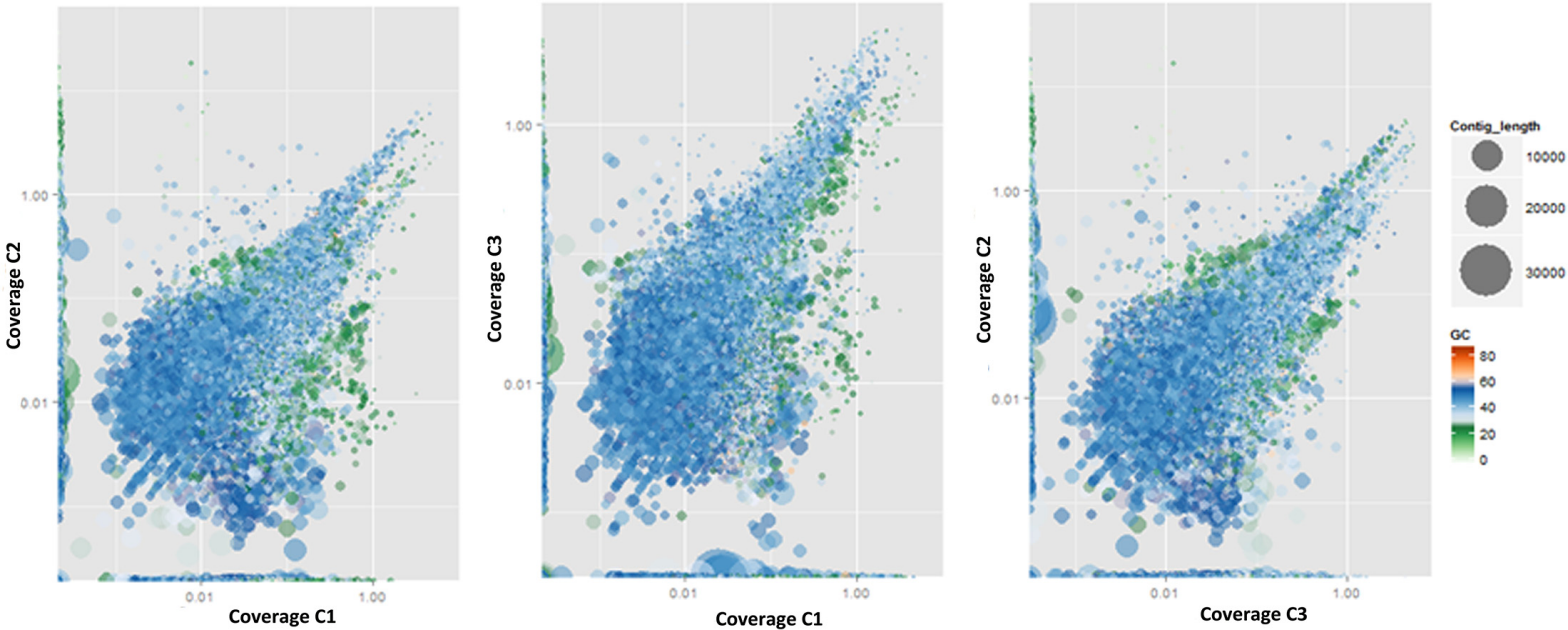
Comparative GC distribution among all the three samples i.e., C1 vs. C2, C1 vs. C3 and C3 vs. C2 is represented as scatter plots. The size of the spot denotes the contig length while the color denotes its GC coverage.

Comparative GC distribution among all the three samples (C1 vs. C2, C1 vs. C3 and C3 vs. C2) revealed GC content of 20–45% (Fig 3). The relative microbial loads for the three samples were calculated by normalizing the number of reads mapping to a contig by the corresponding contig length. This normalized estimate provides us with a new opportunity to understand the relative copy numbers of genetic material between the samples. Normalized output suggests the GC distribution is relatively unaltered by the differences in the number of input reads among the samples considered in the analysis. The various types of microbial communities as well as the comparative microbial load among the three samples are shown in Fig 2. The proportion taxonomic distribution at class level among the datasets C1, C2 and C3 for the kingdoms bacteria, eukaryote and archae is depicted in S1 Fig. The major classes of organisms as revealed by the taxonomic classification were largely present in all the three sets of samples considered.

### Pathway analysis

**Fig 4:**
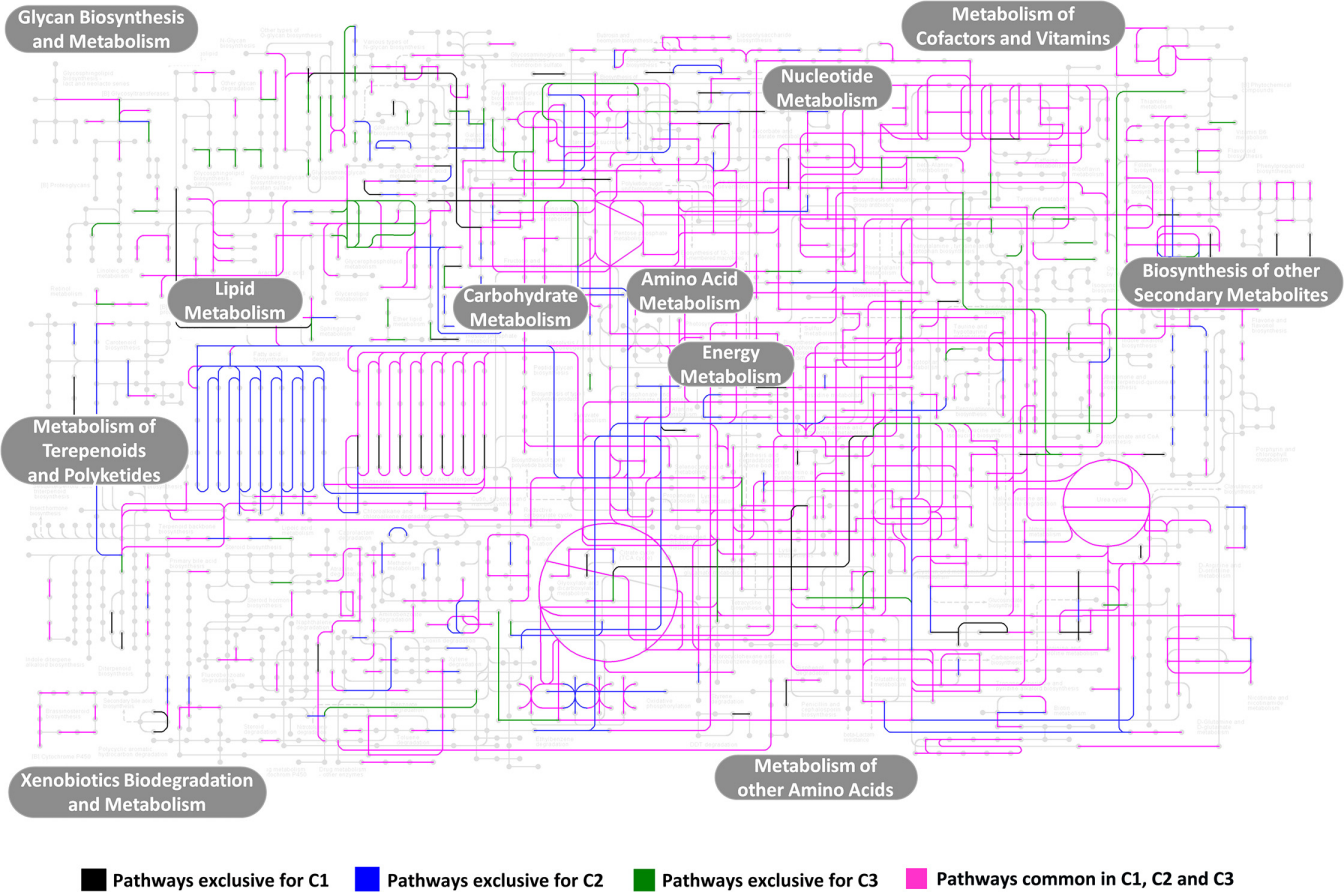
KEGG pathway diagram representing the pathways exclusively present in the three currency datasets, i.e. C1, C2 and C3, as well as the pathways common to all the three datasets. The rectangle boxes (shaded in grey) mention the major pathways enriched in currency metagenome.

The complete list of pathways generated by KEGG Mapper is enlisted in S1 Table. A total of 1556 enzymes mapped to Metabolic Pathways (KO01100). Further analysis revealed 63 enzymes mapped to starch and sucrose metabolism pathway (KO00500) that are involved in cellulose synthesis and degradation. Sample C1 had abundance of 2-dehydropantoate 2-reductase enzyme [E.C.1.1.1.169], which is involved in Pantothenate and CoA biosynthesis, biosynthesis of secondary metabolites and metabolic pathways; sample C2 depicted an abundance of NAD+ synthase enzyme [E.C.6.3.1.5], which is involved in nicotinate and nicotinamide metabolism and metabolic pathways. Sample C3 was abundant in phospholipase D enzyme [E.C.3.1.4.4] involved in glycerophospholipid metabolism, ether lipid metabolism and metabolic pathways. The KEGG pathway analysis for the combined dataset of C1, C2 and C3 revealed common pathways of DNA-directed RNA polymerase enzyme [E.C.2.7.7.6] involved in pyrimidine metabolism, purine metabolism and metabolic pathways associated with core housekeeping functions. The least enriched pathways in the combined dataset included heme-transporting enzyme [EC:3.6.3.41] involved in ABC transporters. Pathways common to all set and exclusive to each set is depicted pictorially in Fig 4.

### Characterization of the pathogens and cellulose degrading organisms

We further analyzed the pathogens, which could be identified using the metagenome assemblies. We specifically analyzed for two sets of organisms: *viz*. pathogens and cellulose degrading organisms. The former was important from a public health point of view, while the latter was relevant in terms of the life of the currency note. To this end, the mappings and assignments from MEGAN were considered and compared with a manually curated list of 1156 pathogenic and 75 cellulose degrading species. 68 bacterial, nine eukaryotic and one viral pathogenic species including *Mycobacterium tuberculosis*, *Plasmodium falciparum*, *Bovine viral diarrhea virus 1* were observed to sojourn on the currency notes at the time of DNA isolation. 11 bacterial pathogens including *Staphylococcus aureus*, *Staphylococcus epidermidis*, *Staphylococcus saprophyticus*, *Staphylococcus haemolyticus*, *Corynebacterium glutamicum*, *Pseudomonas syringae*, *Bacillus cereus*, *Bacillus thuringiensis*, *Bacillus halodurans*, *Bacillus clausii*, *Enterococcus faecalis* were present in all the three samples. Additionally, 14 cellulose degrading bacterial species including *Acidothermus cellulolyticus*, *Cellulomonas flavigena*, *Ruminococcus albus* were found on the currency notes. The complete list of cellulose degrading and pathogenic organisms sojourning on the currency notes is displayed in S2 Table.

### Screening of the metagenome gene repertoire for antibiotic resistance genes

**Fig 5:**
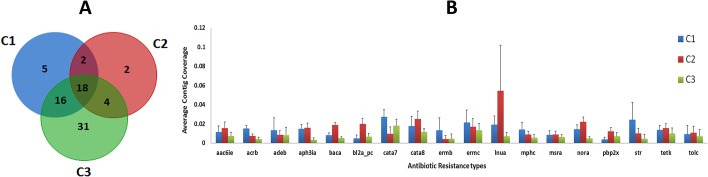
(A): The venn diagram depicts the number of unique and common antibiotic resistance genes between the three samples. (B): Average contig coverage for 18 common antibiotic resistance genes between the three samples is represented as a bar chart. X-axis: The antibiotic resistance genes types and Y-axis: The average contig coverage. C1, C2 and C3 are represented by red, blue and green color bars, respectively. Error bars represent the standard error. [**aac6ie**, Aminoglycoside N-acetyltransferase, (Resistance profile: amikacin, dibekacin, isepamicin, netilmicin, sisomicin, tobramycin); **acrb**, Resistance-nodulation-cell division transporter system- Multidrug resistance efflux pump, (Resistance profile: acriflavin, aminoglycoside, beta_lactam, glycylcycline, macrolide); **adeb**: Resistance-nodulation-cell division transporter system. Multidrug resistance efflux pump, (Resistance profile: aminoglycoside; chloramphenicol); **aph3ia**: Aminoglycoside O-phosphotransferase, (Resistance profile: gentamincin b, kanamycin, lividomycin, neomycin, paromomycin, ribostamycin); **baca**: Undecaprenyl pyrophosphate phosphatase, (Resistance profile: bacitracin); **bl2a_pc**: Class A beta-lactamase, (Resistance profile: penicillin); **cata7**: Group A chloramphenicol acetyltransferase, (Resistance profile: chloramphenicol); **cata8**: Group A chloramphenicol acetyltransferase, (Resistance profile: chloramphenicol); **ermb**: rRNA adenine N-6-methyltransferase, (Resistance profile: lincosamide, macrolide, streptogramin) b; **ermc**: rRNA adenine N-6-methyltransferase, (Resistance profile: lincosamide; macrolide; streptogramin b); **lnua**: Lincosamide nucleotidyltransferase, (Resistance profile: lincomycin; **mphc**: Macrolide phosphotransferase, Resistance profile: macrolide); **msra**: ABC transporter system, Macrolide-Lincosamide-Streptogramin B efflux pump, (Resistance profile: lincosamide; macrolide; streptogramin_b); **nora**: multidrug efflux protein NorA, (Resistance profile: quinolone); **pbp2x**: penicillin-binding protein 2, (Resistance profile: penicillin); **str**: streptomycin 3'-phosphotransferase, (Resistance profile: streptomycin); **tetk**: tetracycline efflux pump, (Resistance profile: tetracycline); **tolc**: Resistance-nodulation-cell division transporter system, (Resistance profile: acriflavin, aminoglycoside, beta_lactam, glycylcycline, macrolide)].

We further attempted to characterize the antibiotic resistance genes of the microbiome derived from currency notes. To this end, we used ARDB, a popular and well curated resource of antibiotic resistance genes [52]. A custom annotation script was used across the contigs to identify potential antibiotic resistance genes in the assembled contigs, which had close similarity with the gene sequences in the entries of the database. Our analysis revealed 347 contigs that had potential matches with the database, encompassing 203 genes and 78 antibiotic resistance types. Comparative analysis revealed 18 out of 78 antibiotic resistance genes to be present in all the three sets of samples considered (Fig 5). S3 Table enlists the complete list of 78 antibiotic resistance genes.

Apart from identifying the pathogens and the antibiotics resistance genes in the metagenome samples, the shotgun approach also provides a unique opportunity to characterize the antibiotic resistance patterns for the pathogens in the metagenome. Such an analysis was aimed to provide an insight into the resistance spectrum of the pathogens, which could be potentially transmitted via fomite. 19 of the 78 pathogens harbored at least one of the 18 common antibiotic resistance genes. Six of the 11 bacterial pathogens common to the three samples had 18 common antibiotic resistance genes thereby indicating a pan-resistance to antibiotics like penicillin, streptomycin, macrolide, etc.

### PCR validation for antibiotic resistance genes

The PCR products from separate collection were checked by running on 1.2% agarose gel. All the samples showed positive result for presence of antibiotic resistance genes *aph3ia* and *baca*.

## Discussion and Conclusions

Fomites as a source of infectious agents have been one of the active areas of research in the recent past [54–56]. Several studies in the recent years have provided insights into the microbiome associated with fomites from a variety of sources [57–59]. Currency notes have been one of the most common exchangeable medium for fomites and have also been extensively evaluated for their ability to transmit pathogenic organisms [60,61]. Many of these studies have relied on culture dependent methods for isolation and analysis of pathogens and enumeration of their drug resistance patterns using regular biochemical techniques [23,62]. In recent reports, where culture independent approaches have been used, the limitation of the approach precluded the identification of drug resistance patterns [60]. The availability of next generation sequencing approaches coupled with computational analysis algorithms have been extensively used in the recent years towards investigating the diversity, characterization and enumeration of their gene content [60–62]. One of the commonly employed approaches have been shotgun metagenome sequencing [63,64]. In the present study, we have performed shotgun sequencing and analysis of the sojourning microbiome of most circulated and least circulated Indian paper currency notes. The eukaryotic species were majorly represented in the contigs but the diversity of the bacteria phyla was noteworthy. The present data largely corroborates with previous observations of the presence of plant spoilage causing fungal genetic material from currency samples [8,62,65]. The diversity of drug resistance genes identified in the present study also uncovers a large resistance to antibiotics belonging to different classes, a subset of which also corroborates with culture dependent studies in the recent past [24]. We also observed that our samples constituted a number of species belonging to pathogenic and cellulose bacterial family. It was found that 14 bacterial, 12 eukaryotic and one viral pathogenic species included species belonging to mycobacteria, corynebacteria, bacillus, etc. 13 cellulose degrading bacterial family included Cellulomonadaceae, Clostridiaceae, Eubacteriaceae, Flavobacteriaceae, etc. which are likely to be responsible for degrading the paper currency notes; thereby reducing their life span in circulation. The presence of cellulose degrading enzymes was also confirmed by the pathway analysis.

The present study is not without caveats. The major caveat being the presence or absence of genetic material would not necessarily affirm to the presence of the live organism. This is especially true in the case of pathogens, where the mere presence of the genetic material from the pathogen would not necessarily mean the sample is infective, though the presence of genetic material would necessarily mean the sample was contaminated at some point in time. The other caveat being that our methodology cannot identify the organisms harboring the antibiotic resistance genes, as many of them are plasmid encoded or horizontally transferred [66]. Nevertheless our approach provides holistic view of the diversity of pathogens associated with currency notes. The present study provides an interesting pipeline for a variety of future applications including bio-surveillance of exchangeable fomites for infectious agents. The methodology could also potentially find application to identify sources for drug resistance genes, and evaluation of sterility and cleanliness of critical areas including operation theatres and medical equipment, and could also serve as an approach to track and identify sources of epidemics. In addition, this method could find potential scope in screening for potentially emerging infectious agents and agents of bioterrorism.

## Supporting Information

S1 Fig Relative taxonomic distribution at class level for the three datasets C1, C2 and C3. The proportion distribution was calculated from the taxonomic classification obtained using Megan. The percentage contribution of each sample set is mentioned inside the pie charts. C1 corresponds to ₹ 10 currency notes, C2 corresponds to ₹ 20 currency notes and C3 corresponds to ₹ 100 currency notes.
(TIF)

S1 Table Complete list of pathways present in our metagenomic data generated by KEGG Mapper. (DOCX)

S2 Table List of cellulolytic bacteria and pathogenic bacteria, virus and eukaryotes present in three datasets C1, C2 and C3. (DOCX)

S3 Table List of 78 antibiotic resistance genes for the three datasets C1, C2 and C3 annotated from the contigs using ARDB. The numbers denotes the number of contigs mapping to each resistant gene.
(DOCX)
