# Supplementary figures and images for: Screening Currency Notes for Microbial Pathogens and Antibiotic Resistance Genes Using a Shotgun Metagenomic Approach
Source: PLoS One. 2015 Jun 2;10(6):e0128711. doi: 10.1371/journal.pone.0128711 (PMC4452720; doi:10.1371/journal.pone.0128711)

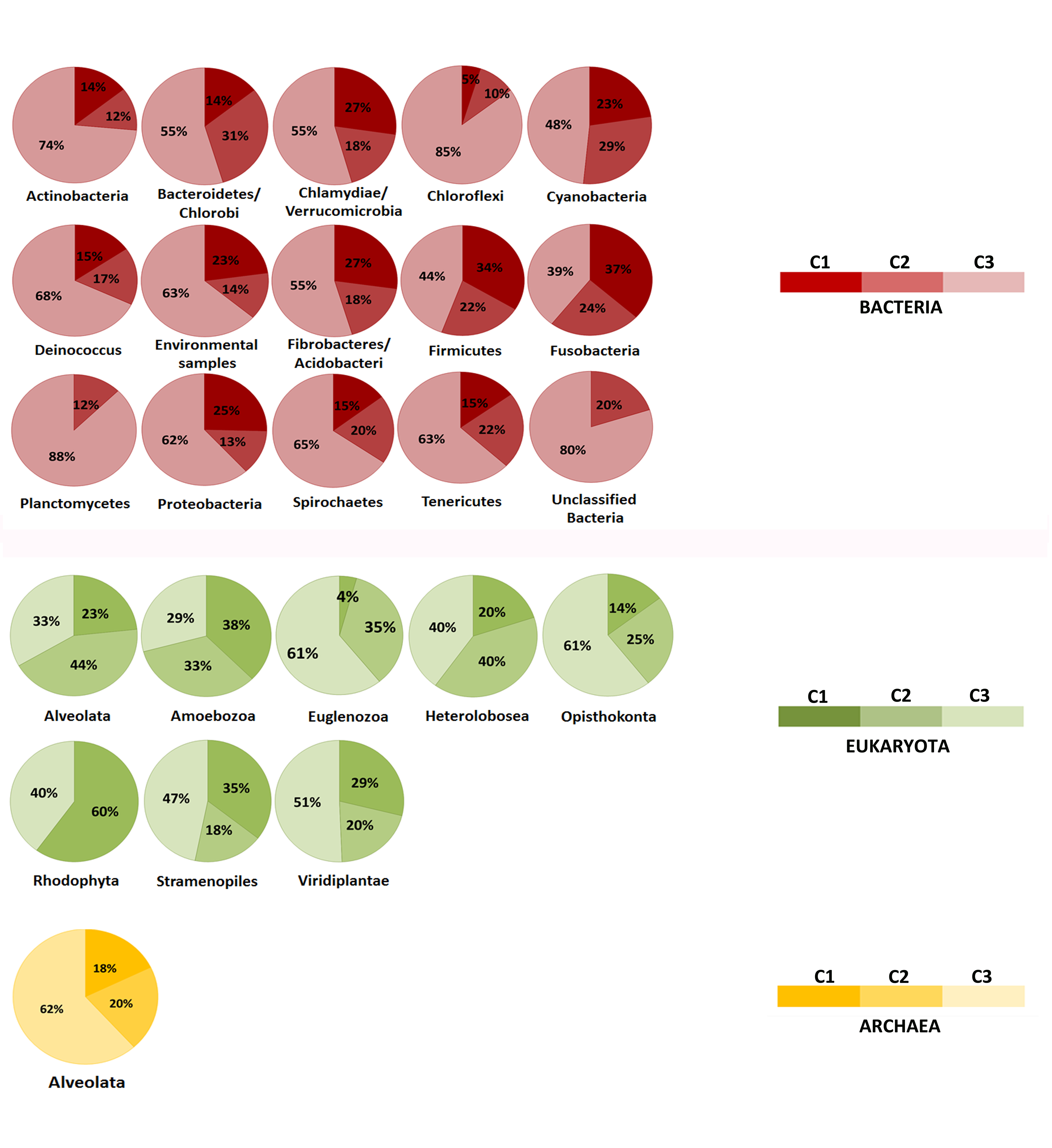

Supplement: S1 Fig — The proportion distribution was calculated from the taxonomic classification obtained using Megan. The percentage contribution of each sample set is mentioned inside the pie charts. C1 corresponds to ₹ 10 currency notes, C2 corresponds to ₹ 20 currency notes and C3 corresponds to ₹ 100 currency notes. (TIF) [file pone.0128711.s001.tif]
