# Supplementary material for: Screening Currency Notes for Microbial Pathogens and Antibiotic Resistance Genes Using a Shotgun Metagenomic Approach
Source: PLoS One. 2015 Jun 2;10(6):e0128711. doi: 10.1371/journal.pone.0128711 (PMC4452720; doi:10.1371/journal.pone.0128711)
Supplement: S1 Table — (DOCX) [file pone.0128711.s002.docx]

| **Table S1**: Complete list of pathways present in our metagenomic data generated by KEGG Mapper | | |
| --- | --- | --- |
| **S.No.** | **Pathway ID** | **Pathway Name** |
| 1 | ko01100 | Metabolic pathways (1556) |
| 2 | ko01110 | Biosynthesis of secondary metabolites (619) |
| 3 | ko01120 | Microbial metabolism in diverse environments (510) |
| 4 | ko01230 | Biosynthesis of amino acids (233) |
| 5 | ko00230 | Purine metabolism (199) |
| 6 | ko01200 | Carbon metabolism (185) |
| 7 | ko02020 | Two-component system (144) |
| 8 | ko00240 | Pyrimidine metabolism (124) |
| 9 | ko00330 | Arginine and proline metabolism (98) |
| 10 | ko01220 | Degradation of aromatic compounds (95) |
| 11 | ko00190 | Oxidative phosphorylation (94) |
| 12 | ko00400 | Phenylalanine, tyrosine and tryptophan biosynthesis (78) |
| 13 | ko01212 | Fatty acid metabolism (76) |
| 14 | ko00520 | Amino sugar and nucleotide sugar metabolism (75) |
| 15 | ko00564 | Glycerophospholipid metabolism (73) |
| 16 | ko00720 | Carbon fixation pathways in prokaryotes (72) |
| 17 | ko00620 | Pyruvate metabolism (72) |
| 18 | ko04010 | MAPK signaling pathway (70) |
| 19 | ko00260 | Glycine, serine and threonine metabolism (69) |
| 20 | ko04120 | Ubiquitin mediated proteolysis (69) |
| 21 | ko00640 | Propanoate metabolism (67) |
| 22 | ko00010 | Glycolysis / Gluconeogenesis (66) |
| 23 | ko00860 | Porphyrin and chlorophyll metabolism (66) |
| 24 | ko00650 | Butanoate metabolism (66) |
| 25 | ko01210 | 2-Oxocarboxylic acid metabolism (66) |
| 26 | ko00270 | Cysteine and methionine metabolism (65) |
| 27 | ko00280 | Valine, leucine and isoleucine degradation (64) |
| 28 | ko00500 | Starch and sucrose metabolism (63) |
| 29 | ko00051 | Fructose and mannose metabolism (63) |
| 30 | ko05200 | Pathways in cancer (61) |
| 31 | ko00310 | Lysine degradation (60) |
| 32 | ko00071 | Fatty acid degradation (59) |
| 33 | ko00030 | Pentose phosphate pathway (58) |
| 34 | ko00362 | Benzoate degradation (55) |
| 35 | ko00250 | Alanine, aspartate and glutamate metabolism (55) |
| 36 | ko00680 | Methane metabolism (51) |
| 37 | ko02010 | ABC transporters (50) |
| 38 | ko04014 | Ras signaling pathway (49) |
| 39 | ko04151 | PI3K-Akt signaling pathway (48) |
| 40 | ko00380 | Tryptophan metabolism (47) |
| 41 | ko00561 | Glycerolipid metabolism (47) |
| 42 | ko02060 | Phosphotransferase system (PTS) (46) |
| 43 | ko00052 | Galactose metabolism (45) |
| 44 | ko00630 | Glyoxylate and dicarboxylate metabolism (45) |
| 45 | ko00350 | Tyrosine metabolism (44) |
| 46 | ko05169 | Epstein-Barr virus infection (44) |
| 47 | ko00300 | Lysine biosynthesis (42) |
| 48 | ko04015 | Rap1 signaling pathway (42) |
| 49 | ko05166 | HTLV-I infection (42) |
| 50 | ko00627 | Aminobenzoate degradation (41) |
| 51 | ko00020 | Citrate cycle (TCA cycle) (40) |
| 52 | ko04068 | FoxO signaling pathway (40) |
| 53 | ko05206 | MicroRNAs in cancer (39) |
| 54 | ko00360 | Phenylalanine metabolism (39) |
| 55 | ko04722 | Neurotrophin signaling pathway (38) |
| 56 | ko03410 | Base excision repair (38) |
| 57 | ko00910 | Nitrogen metabolism (38) |
| 58 | ko00670 | One carbon pool by folate (37) |
| 59 | ko03030 | DNA replication (37) |
| 60 | ko04921 | Oxytocin signaling pathway (37) |
| 61 | ko00970 | Aminoacyl-tRNA biosynthesis (37) |
| 62 | ko04270 | Vascular smooth muscle contraction (36) |
| 63 | ko05205 | Proteoglycans in cancer (36) |
| 64 | ko04146 | Peroxisome (36) |
| 65 | ko04144 | Endocytosis (35) |
| 66 | ko04510 | Focal adhesion (35) |
| 67 | ko04020 | Calcium signaling pathway (35) |
| 68 | ko01040 | Biosynthesis of unsaturated fatty acids (35) |
| 69 | ko00900 | Terpenoid backbone biosynthesis (35) |
| 70 | ko04022 | cGMP-PKG signaling pathway (35) |
| 71 | ko04062 | Chemokine signaling pathway (34) |
| 72 | ko04141 | Protein processing in endoplasmic reticulum (34) |
| 73 | ko00550 | Peptidoglycan biosynthesis (33) |
| 74 | ko00130 | Ubiquinone and other terpenoid-quinone biosynthesis (33) |
| 75 | ko05203 | Viral carcinogenesis (33) |
| 76 | ko04660 | T cell receptor signaling pathway (33) |
| 77 | ko05012 | Parkinson's disease (33) |
| 78 | ko04024 | cAMP signaling pathway (32) |
| 79 | ko04912 | GnRH signaling pathway (32) |
| 80 | ko00513 | Various types of N-glycan biosynthesis (32) |
| 81 | ko00710 | Carbon fixation in photosynthetic organisms (32) |
| 82 | ko00830 | Retinol metabolism (32) |
| 83 | ko00061 | Fatty acid biosynthesis (32) |
| 84 | ko04360 | Axon guidance (32) |
| 85 | ko04110 | Cell cycle (32) |
| 86 | ko04810 | Regulation of actin cytoskeleton (32) |
| 87 | ko05164 | Influenza A (31) |
| 88 | ko00790 | Folate biosynthesis (31) |
| 89 | ko04012 | ErbB signaling pathway (31) |
| 90 | ko00920 | Sulfur metabolism (31) |
| 91 | ko04611 | Platelet activation (31) |
| 92 | ko00760 | Nicotinate and nicotinamide metabolism (31) |
| 93 | ko00480 | Glutathione metabolism (31) |
| 94 | ko04750 | Inflammatory mediator regulation of TRP channels (31) |
| 95 | ko00410 | beta-Alanine metabolism (31) |
| 96 | ko04910 | Insulin signaling pathway (31) |
| 97 | ko04111 | Cell cycle - yeast (30) |
| 98 | ko03440 | Homologous recombination (30) |
| 99 | ko04914 | Progesterone-mediated oocyte maturation (30) |
| 100 | ko00562 | Inositol phosphate metabolism (29) |
| 101 | ko00540 | Lipopolysaccharide biosynthesis (29) |
| 102 | ko05010 | Alzheimer's disease (29) |
| 103 | ko01057 | Biosynthesis of type II polyketide products (29) |
| 104 | ko05230 | Central carbon metabolism in cancer (29) |
| 105 | ko00600 | Sphingolipid metabolism (28) |
| 106 | ko00040 | Pentose and glucuronateinterconversions (28) |
| 107 | ko04114 | Oocyte meiosis (28) |
| 108 | ko03018 | RNA degradation (28) |
| 109 | ko04540 | Gap junction (28) |
| 110 | ko00340 | Histidine metabolism (28) |
| 111 | ko04550 | Signaling pathways regulating pluripotency of stem cells (27) |
| 112 | ko04932 | Non-alcoholic fatty liver disease (NAFLD) (26) |
| 113 | ko04066 | HIF-1 signaling pathway (26) |
| 114 | ko04113 | Meiosis - yeast (26) |
| 115 | ko05161 | Hepatitis B (26) |
| 116 | ko05168 | Herpes simplex infection (26) |
| 117 | ko03020 | RNA polymerase (26) |
| 118 | ko05204 | Chemical carcinogenesis (26) |
| 119 | ko04380 | Osteoclast differentiation (26) |
| 120 | ko05162 | Measles (26) |
| 121 | ko04668 | TNF signaling pathway (25) |
| 122 | ko00770 | Pantothenate and CoA biosynthesis (25) |
| 123 | ko03008 | Ribosome biogenesis in eukaryotes (25) |
| 124 | ko05160 | Hepatitis C (24) |
| 125 | ko03430 | Mismatch repair (24) |
| 126 | ko04919 | Thyroid hormone signaling pathway (24) |
| 127 | ko04064 | NF-kappa B signaling pathway (24) |
| 128 | ko04070 | Phosphatidylinositol signaling system (24) |
| 129 | ko00590 | Arachidonic acid metabolism (24) |
| 130 | ko05215 | Prostate cancer (23) |
| 131 | ko00361 | Chlorocyclohexane and chlorobenzene degradation (23) |
| 132 | ko05152 | Tuberculosis (23) |
| 133 | ko03013 | RNA transport (23) |
| 134 | ko00623 | Toluene degradation (23) |
| 135 | ko00514 | Other types of O-glycan biosynthesis (22) |
| 136 | ko04520 | Adherens junction (22) |
| 137 | ko00740 | Riboflavin metabolism (22) |
| 138 | ko00626 | Naphthalene degradation (22) |
| 139 | ko00980 | Metabolism of xenobiotics by cytochrome P450(22) |
| 140 | ko04726 | Serotonergic synapse (22) |
| 141 | ko00450 | Selenocompound metabolism (22) |
| 142 | ko05016 | Huntington's disease (21) |
| 143 | ko05202 | Transcriptional misregulation in cancer (21) |
| 144 | ko05034 | Alcoholism (21) |
| 145 | ko04915 | Estrogen signaling pathway (21) |
| 146 | ko00062 | Fatty acid elongation (21) |
| 147 | ko04970 | Salivary secretion (20) |
| 148 | ko04666 | Fc gamma R-mediated phagocytosis (20) |
| 149 | ko04916 | Melanogenesis (20) |
| 150 | ko00565 | Ether lipid metabolism (20) |
| 151 | ko04713 | Circadian entrainment (20) |
| 152 | ko00053 | Ascorbate and aldarate metabolism (20) |
| 153 | ko00140 | Steroid hormone biosynthesis (20) |
| 154 | ko04261 | Adrenergic signaling in cardiomyocytes (20) |
| 155 | ko03050 | Proteasome (20) |
| 156 | ko04152 | AMPK signaling pathway (20) |
| 157 | ko04060 | Cytokine-cytokine receptor interaction (20) |
| 158 | ko03320 | PPAR signaling pathway (20) |
| 159 | ko05120 | Epithelial cell signaling in Helicobacter pylori infection (20) |
| 160 | ko04913 | Ovarian steroidogenesis (20) |
| 161 | ko04725 | Cholinergic synapse (20) |
| 162 | ko00982 | Drug metabolism - cytochrome P450 (19) |
| 163 | ko00622 | Xylene degradation (19) |
| 164 | ko00281 | Geraniol degradation (19) |
| 165 | ko04620 | Toll-like receptor signaling pathway (19) |
| 166 | ko05220 | Chronic myeloid leukemia (19) |
| 167 | ko04664 | Fc epsilon RI signaling pathway (19) |
| 168 | ko05214 | Glioma (19) |
| 169 | ko00591 | Linoleic acid metabolism (19) |
| 170 | ko05145 | Toxoplasmosis (19) |
| 171 | ko00290 | Valine, leucine and isoleucine biosynthesis (18) |
| 172 | ko00510 | N-Glycan biosynthesis (18) |
| 173 | ko00930 | Caprolactam degradation (18) |
| 174 | ko00960 | Tropane, piperidine and pyridine alkaloid biosynthesis (18) |
| 175 | ko04370 | VEGF signaling pathway (18) |
| 176 | ko04971 | Gastric acid secretion (18) |
| 177 | ko04650 | Natural killer cell mediated cytotoxicity (18) |
| 178 | ko00624 | Polycyclic aromatic hydrocarbon degradation (18) |
| 179 | ko03460 | Fanconi anemia pathway (18) |
| 180 | ko00592 | alpha-Linolenic acid metabolism (18) |
| 181 | ko04724 | Glutamatergic synapse (18) |
| 182 | ko04210 | Apoptosis (18) |
| 183 | ko04972 | Pancreatic secretion (18) |
| 184 | ko04142 | Lysosome (18) |
| 185 | ko05211 | Renal cell carcinoma (17) |
| 186 | ko04723 | Retrograde endocannabinoid signaling (17) |
| 187 | ko05218 | Melanoma (17) |
| 188 | ko04727 | GABAergic synapse (17) |
| 189 | ko05142 | Chagas disease (American trypanosomiasis) (17) |
| 190 | ko04720 | Long-term potentiation (17) |
| 191 | ko05032 | Morphine addiction (17) |
| 192 | ko04918 | Thyroid hormone synthesis (17) |
| 193 | ko04622 | RIG-I-like receptor signaling pathway (17) |
| 194 | ko04310 | Wnt signaling pathway (17) |
| 195 | ko04530 | Tight junction (16) |
| 196 | ko05212 | Pancreatic cancer (16) |
| 197 | ko04350 | TGF-beta signaling pathway (16) |
| 198 | ko00253 | Tetracycline biosynthesis (16) |
| 199 | ko03040 | Spliceosome (16) |
| 200 | ko00730 | Thiamine metabolism (16) |
| 201 | ko00983 | Drug metabolism - other enzymes (16) |
| 202 | ko04115 | p53 signaling pathway (16) |
| 203 | ko00780 | Biotin metabolism (16) |
| 204 | ko00460 | Cyanoamino acid metabolism (16) |
| 205 | ko00625 | Chloroalkane and chloroalkene degradation (16) |
| 206 | ko00601 | Glycosphingolipid biosynthesis - lacto and neolacto series (16) |
| 207 | ko00903 | Limonene and pinene degradation (16) |
| 208 | ko04730 | Long-term depression (16) |
| 209 | ko05223 | Non-small cell lung cancer (16) |
| 210 | ko04626 | Plant-pathogen interaction (15) |
| 211 | ko04728 | Dopaminergic synapse (15) |
| 212 | ko04976 | Bile secretion (15) |
| 213 | ko04662 | B cell receptor signaling pathway (15) |
| 214 | ko00100 | Steroid biosynthesis (15) |
| 215 | ko00312 | beta-Lactam resistance (15) |
| 216 | ko00940 | Phenylpropanoid biosynthesis (15) |
| 217 | ko04911 | Insulin secretion (15) |
| 218 | ko03420 | Nucleotide excision repair (15) |
| 219 | ko04630 | Jak-STAT signaling pathway (14) |
| 220 | ko05221 | Acute myeloid leukemia (14) |
| 221 | ko00643 | Styrene degradation (14) |
| 222 | ko00534 | Glycosaminoglycan biosynthesis - heparan sulfate / heparin (14) |
| 223 | ko05219 | Bladder cancer (14) |
| 224 | ko00430 | Taurine and hypotaurine metabolism (14) |
| 225 | ko00533 | Glycosaminoglycan biosynthesis - keratan sulfate (13) |
| 226 | ko04075 | Plant hormone signal transduction (13) |
| 227 | ko04011 | MAPK signaling pathway - yeast (13) |
| 228 | ko00660 | C5-Branched dibasic acid metabolism (13) |
| 229 | ko01056 | Biosynthesis of type II polyketide backbone (13) |
| 230 | ko05213 | Endometrial cancer (13) |
| 231 | ko00950 | Isoquinoline alkaloid biosynthesis (13) |
| 232 | ko00401 | Novobiocin biosynthesis (13) |
| 233 | ko00511 | Other glycan degradation (13) |
| 234 | ko05133 | Pertussis (12) |
| 235 | ko04917 | Prolactin signaling pathway (12) |
| 236 | ko04920 | Adipocytokine signaling pathway (12) |
| 237 | ko04390 | Hippo signaling pathway (12) |
| 238 | ko04670 | Leukocyte transendothelial migration (11) |
| 239 | ko04150 | mTOR signaling pathway (11) |
| 240 | ko05414 | Dilated cardiomyopathy (11) |
| 241 | ko00563 | Glycosylphosphatidylinositol(GPI)-anchor biosynthesis (11) |
| 242 | ko05222 | Small cell lung cancer (11) |
| 243 | ko05140 | Leishmaniasis (11) |
| 244 | ko04623 | Cytosolic DNA-sensing pathway (11) |
| 245 | ko00791 | Atrazine degradation (10) |
| 246 | ko00532 | Glycosaminoglycan biosynthesis - chondroitin sulfate / dermatan sulfate (10) |
| 247 | ko03015 | mRNA surveillance pathway (10) |
| 248 | ko05210 | Colorectal cancer (10) |
| 249 | ko00633 | Nitrotoluene degradation (10) |
| 250 | ko05131 | Shigellosis (10) |
| 251 | ko01053 | Biosynthesis of siderophore group nonribosomal peptides (10) |
| 252 | ko00906 | Carotenoid biosynthesis (10) |
| 253 | ko05132 | Salmonella infection (10) |
| 254 | ko03450 | Non-homologous end-joining (9) |
| 255 | ko04112 | Cell cycle - Caulobacter (9) |
| 256 | ko00332 | Carbapenem biosynthesis (9) |
| 257 | ko05014 | Amyotrophic lateral sclerosis (ALS) (9) |
| 258 | ko00521 | Streptomycin biosynthesis (9) |
| 259 | ko04930 | Type II diabetes mellitus (9) |
| 260 | ko00621 | Dioxin degradation (9) |
| 261 | ko04122 | Sulfur relay system (9) |
| 262 | ko05031 | Amphetamine addiction (9) |
| 263 | ko04960 | Aldosterone-regulated sodium reabsorption (8) |
| 264 | ko04340 | Hedgehog signaling pathway (8) |
| 265 | ko05110 | Vibrio cholerae infection (8) |
| 266 | ko05111 | Vibrio cholerae pathogenic cycle (8) |
| 267 | ko04140 | Regulation of autophagy (8) |
| 268 | ko05340 | Primary immunodeficiency (8) |
| 269 | ko00364 | Fluorobenzoate degradation (8) |
| 270 | ko03060 | Protein export (8) |
| 271 | ko00403 | Indolediterpene alkaloid biosynthesis (8) |
| 272 | ko00750 | Vitamin B6 metabolism (8) |
| 273 | ko00072 | Synthesis and degradation of ketone bodies (8) |
| 274 | ko04621 | NOD-like receptor signaling pathway (8) |
| 275 | ko00966 | Glucosinolate biosynthesis (7) |
| 276 | ko00073 | Cutin, suberine and wax biosynthesis (7) |
| 277 | ko05020 | Prion diseases (7) |
| 278 | ko00905 | Brassinosteroid biosynthesis (7) |
| 279 | ko04961 | Endocrine and other factor-regulated calcium reabsorption (7) |
| 280 | ko05146 | Amoebiasis (7) |
| 281 | ko04975 | Fat digestion and absorption (7) |
| 282 | ko05150 | Staphylococcus aureus infection (7) |
| 283 | ko00363 | Bisphenol degradation (7) |
| 284 | ko04964 | Proximal tubule bicarbonate reclamation (7) |
| 285 | ko00604 | Glycosphingolipid biosynthesis - ganglio series (7) |
| 286 | ko04745 | Phototransduction - fly (6) |
| 287 | ko00311 | Penicillin and cephalosporin biosynthesis (6) |
| 288 | ko00603 | Glycosphingolipid biosynthesis - globo series (6) |
| 289 | ko04391 | Hippo signaling pathway - fly (6) |
| 290 | ko00642 | Ethylbenzene degradation (6) |
| 291 | ko05143 | African trypanosomiasis (6) |
| 292 | ko04013 | MAPK signaling pathway - fly (6) |
| 293 | ko05216 | Thyroid cancer (6) |
| 294 | ko05100 | Bacterial invasion of epithelial cells (6) |
| 295 | ko00232 | Caffeine metabolism (5) |
| 296 | ko04973 | Carbohydrate digestion and absorption (5) |
| 297 | ko03070 | Bacterial secretion system (5) |
| 298 | ko04962 | Vasopressin-regulated water reabsorption (5) |
| 299 | ko04145 | Phagosome (5) |
| 300 | ko04978 | Mineral absorption (5) |
| 301 | ko04330 | Notch signaling pathway (5) |
| 302 | ko00440 | Phosphonate and phosphinate metabolism (5) |
| 303 | ko02030 | Bacterial chemotaxis (5) |
| 304 | ko01051 | Biosynthesis of ansamycins (5) |
| 305 | ko00512 | Mucin type O-Glycan biosynthesis (5) |
| 306 | ko04974 | Protein digestion and absorption (5) |
| 307 | ko01052 | Type I polyketide structures (5) |
| 308 | ko00231 | Puromycin biosynthesis (5) |
| 309 | ko00471 | D-Glutamine and D-glutamate metabolism (5) |
| 310 | ko00120 | Primary bile acid biosynthesis (5) |
| 311 | ko05130 | Pathogenic Escherichia coli infection (4) |
| 312 | ko04614 | Renin-angiotensin system (4) |
| 313 | ko04610 | Complement and coagulation cascades (4) |
| 314 | ko04640 | Hematopoietic cell lineage (4) |
| 315 | ko05134 | Legionellosis (4) |
| 316 | ko04740 | Olfactory transduction (4) |
| 317 | ko04744 | Phototransduction (4) |
| 318 | ko03022 | Basal transcription factors (4) |
| 319 | ko04710 | Circadian rhythm (4) |
| 320 | ko00965 | Betalain biosynthesis (4) |
| 321 | ko04742 | Taste transduction (4) |
| 322 | ko05323 | Rheumatoid arthritis (4) |
| 323 | ko04966 | Collecting duct acid secretion (4) |
| 324 | ko00531 | Glycosaminoglycan degradation (4) |
| 325 | ko05030 | Cocaine addiction (4) |
| 326 | ko00523 | Polyketide sugar unit biosynthesis (4) |
| 327 | ko05410 | Hypertrophic cardiomyopathy (HCM) (3) |
| 328 | ko00522 | Biosynthesis of 12-, 14- and 16-membered macrolides (3) |
| 329 | ko04320 | Dorso-ventral axis formation (3) |
| 330 | ko04260 | Cardiac muscle contraction (3) |
| 331 | ko04514 | Cell adhesion molecules (CAMs) (3) |
| 332 | ko00351 | DDT degradation (3) |
| 333 | ko04940 | Type I diabetes mellitus (3) |
| 334 | ko04721 | Synaptic vesicle cycle (3) |
| 335 | ko00473 | D-Alanine metabolism (3) |
| 336 | ko05416 | Viral myocarditis (3) |
| 337 | ko00524 | Butirosin and neomycin biosynthesis (3) |
| 338 | ko04711 | Circadian rhythm - fly (3) |
| 339 | ko00909 | Sesquiterpenoid and triterpenoid biosynthesis (2) |
| 340 | ko00945 | Stilbenoid, diarylheptanoid and gingerol biosynthesis (2) |
| 341 | ko05217 | Basal cell carcinoma (2) |
| 342 | ko00785 | Lipoic acid metabolism (2) |
| 343 | ko00984 | Steroid degradation (2) |
| 344 | ko00196 | Photosynthesis - antenna proteins (2) |
| 345 | ko00942 | Anthocyanin biosynthesis (2) |
| 346 | ko00981 | Insect hormone biosynthesis (2) |
| 347 | ko04712 | Circadian rhythm - plant (2) |
| 348 | ko00195 | Photosynthesis (2) |
| 349 | ko04080 | Neuroactive ligand-receptor interaction (2) |
| 350 | ko04950 | Maturity onset diabetes of the young (2) |
| 351 | ko00254 | Aflatoxin biosynthesis (2) |
| 352 | ko04612 | Antigen processing and presentation (2) |
| 353 | ko05310 | Asthma (2) |
| 354 | ko00908 | Zeatin biosynthesis (2) |
| 355 | ko04977 | Vitamin digestion and absorption (2) |
| 356 | ko00901 | Indole alkaloid biosynthesis (1) |
| 357 | ko00943 | Isoflavonoid biosynthesis (1) |
| 358 | ko01055 | Biosynthesis of vancomycin group antibiotics (1) |
| 359 | ko00904 | Diterpenoid biosynthesis (1) |
| 360 | ko04672 | Intestinal immune network for IgA production (1) |
| 361 | ko00941 | Flavonoid biosynthesis (1) |
| 362 | ko05322 | Systemic lupus erythematosus (1) |
| 363 | ko00944 | Flavone and flavonol biosynthesis (1) |
| 364 | ko05144 | Malaria (1) |
| 365 | ko02040 | Flagellar assembly (1) |
| 366 | ko04512 | ECM-receptor interaction (1) |
|  |  |  |
