# Supplementary material for: Screening Currency Notes for Microbial Pathogens and Antibiotic Resistance Genes Using a Shotgun Metagenomic Approach
Source: PLoS One. 2015 Jun 2;10(6):e0128711. doi: 10.1371/journal.pone.0128711 (PMC4452720; doi:10.1371/journal.pone.0128711)
Supplement: S2 Table — (DOCX) [file pone.0128711.s003.docx]

| **Table S2:** List of cellulolytic bacteria and pathogenic bacteria, virus and eukaryotes present in three datasets C1, C2 and C3 | | | |
| --- | --- | --- | --- |
| **Cellulolytic bacteria** | **Bacterial Pathogens** | **Viral Pathogens** | **Eukaryotic Pathogens** |
| *Achromobacter piechaudii* | *Aeromonas hydrophila* | *Bovine viral diarrhea virus 1* | *Cryptosporidium parvum* |
| *Acidothermus cellulolyticus* | *Bacillus anthracis* |  | *Eimeria tenella* |
| *Cellulomonas flavigena* | *Bacillus cereus* |  | *Entamoeba histolytica* |
| *Clostridium acetobutylicum* | *Bacillus clausii* |  | *Leishmania braziliensis* |
| *Clostridium cellulolyticum* | *Bacillus halodurans* |  | *Plasmodium falciparum* |
| *Clostridium cellulovorans* | *Bacillus licheniformis* |  | *Plasmodium vivax* |
| *Clostridium papyrosolvens* | *Bacillus subtilis* |  | *Plasmodium yoelii* |
| *Clostridium thermocellum* | *Bacillus thuringiensis* |  | *Theileria annulata* |
| *Fibrobacter succinogenes* | *Bdellovibrio bacteriovorus* |  | *Trypanosoma cruzi* |
| *Pseudomonas fluorescens* | *Bordetella avium* |  | *Cryptosporidium parvum* |
| *Pseudomonas mendocina* | *Corynebacterium efficiens* |  | *Eimeria tenella* |
| *Ruminococcus albus* | *Corynebacterium glutamicum* |  | *Entamoeba histolytica* |
| *Thermobifida fusca* | *Corynebacterium jeikeium* |  | *Leishmania braziliensis* |
|  | *Enterococcus faecalis* |  | *Plasmodium falciparum* |
|  | *Escherichia coli* |  | *Plasmodium vivax* |
|  | *Helicobacter pylori* |  |  |
|  | *Listeria innocua* |  |  |
|  | *Listeria ivanovii* |  |  |
|  | *Listeria monocytogenes* |  |  |
|  | *Listeria seeligeri* |  |  |
|  | *Mycobacterium abscessus* |  |  |
|  | *Mycobacterium avium* |  |  |
|  | *Mycobacterium bovis* |  |  |
|  | *Mycobacterium marinum* |  |  |
|  | *Mycobacterium smegmatis* |  |  |
|  | *Mycobacterium tuberculosis* |  |  |
|  | *Mycoplasma penetrans* |  |  |
|  | *Neisseria meningitidis* |  |  |
|  | *Nocardia farcinica* |  |  |
|  | *Pasteurella multocida* |  |  |
|  | *Propionibacterium acnes* |  |  |
|  | *Providencia stuartii* |  |  |
|  | *Pseudomonas aeruginosa* |  |  |
|  | *Pseudomonas entomophila* |  |  |
|  | *Pseudomonas fluorescens* |  |  |
|  | *Pseudomonas mendocina* |  |  |
|  | *Pseudomonas stutzeri* |  |  |
|  | *Pseudomonas syringae* |  |  |
|  | *Ralstonia solanacearum* |  |  |
|  | *Salmonella enterica* |  |  |
|  | *Staphylococcus aureus* |  |  |
|  | *Staphylococcus epidermidis* |  |  |
|  | *Staphylococcus haemolyticus* |  |  |
|  | *Staphylococcus saprophyticus* |  |  |
|  | *Stenotrophomonas maltophilia* |  |  |
|  | *Streptococcus agalactiae* |  |  |
|  | *Streptococcus mutans* |  |  |
|  | *Streptococcus pneumoniae* |  |  |
|  | *Streptococcus pyogenes* |  |  |
|  | *Streptococcus suis* |  |  |
|  | *Streptococcus thermophilus* |  |  |
|  | *Vibrio cholerae* |  |  |
|  | *Vibrio parahaemolyticus* |  |  |
|  | *Vibrio vulnificus* |  |  |
|  | *Yersinia enterocolitica* |  |  |
