# Supplementary material for: Screening Currency Notes for Microbial Pathogens and Antibiotic Resistance Genes Using a Shotgun Metagenomic Approach
Source: PLoS One. 2015 Jun 2;10(6):e0128711. doi: 10.1371/journal.pone.0128711 (PMC4452720; doi:10.1371/journal.pone.0128711)
Supplement: S3 Table — The numbers denotes the number of contigs mapping to each resistant gene. (DOCX) [file pone.0128711.s004.docx]

| **Table S3**: List of 78 antibiotic resistant genes for the three datasets C1, C2 and C3 annotated from the contigs using ARDB. The numbers denotes the count of contigs mapping to each resistant gene. | | | | | | |
| --- | --- | --- | --- | --- | --- | --- |
| **S. No.** | **Resistant Genes** | **Description** | **Resistance Profile** | **C1** | **C2** | **C3** |
| 1 | *aac6ie* | Aminoglycoside N-acetyltransferase, which modifies aminoglycosides by acetylation. | tobramycin netilmicin isepamicin amikacin dibekacin sisomicin | 2 | 1 | 4 |
| 2 | *aadd* | Aminoglycoside O-nucleotidylyltransferase, which modifies aminoglycosides by adenylylation. | tobramycin kanamycin | 1 | 0 | 1 |
| 3 | *acra* | Resistance-nodulation-cell division transporter system. Multidrug resistance efflux pump. | aminoglycoside glycylcycline beta_lactam macrolide acriflavin | 0 | 1 | 3 |
| 4 | *acrb* | Resistance-nodulation-cell division transporter system. Multidrug resistance efflux pump. | aminoglycoside glycylcycline beta_lactam macrolide acriflavin | 11 | 4 | 9 |
| 5 | *adeb* | Resistance-nodulation-cell division transporter system. Multidrug resistance efflux pump. | chloramphenicol aminoglycoside | 1 | 1 | 1 |
| 6 | *adec* | Resistance-nodulation-cell division transporter system. Multidrug resistance efflux pump. | chloramphenicol aminoglycoside | 1 | 0 | 2 |
| 7 | *ant2ia* | Aminoglycoside O-nucleotidylyltransferase, which modifies aminoglycosides by adenylylation. | tobramycin gentamicin dibekacin sisomicin kanamycin | 0 | 0 | 2 |
| 8 | *ant3ia* | Aminoglycoside O-nucleotidylyltransferase, which modifies aminoglycosides by adenylylation. | spectinomycin streptomycin | 0 | 0 | 1 |
| 9 | *aph33ib* | Aminoglycoside O-phosphotransferase, which modifies aminoglycosides by phosphorylation. | streptomycin | 1 | 0 | 4 |
| 10 | *aph3ia* | Aminoglycoside O-phosphotransferase, which modifies aminoglycosides by phosphorylation. | paromomycin kanamycin neomycin ribostamycin lividomycin gentamincin_b | 3 | 1 | 5 |
| 11 | *aph3iiia* | Aminoglycoside O-phosphotransferase, which modifies aminoglycosides by phosphorylation. | paromomycin kanamycin neomycin ribostamycin lividomycin isepamicin butirosin amikacin gentamincin_b | 0 | 2 | 0 |
| 12 | *aph6id* | Aminoglycoside O-phosphotransferase, which modifies aminoglycosides by phosphorylation. | streptomycin | 3 | 0 | 4 |
| 13 | *arna* | Bifunctional enzyme that catalyzes the oxidative decarboxylation of UDP-glucuronic acid (UDP-GlcUA) to UDP-4-keto-arabinose (UDP-Ara4O) and the addition of a formyl group to UDP-4-amino-4-deoxy-L-arabinose (UDP-L-Ara4N) to form UDP-L-4-formamido-arabinose (UDP-L-Ara4FN). The modified arabinose is attached to lipid A and is required for resistance to polymyxin and cationic antimicrobial peptides. | polymyxin | 1 | 0 | 1 |
| 14 | *baca* | Undecaprenyl pyrophosphate phosphatase, which consists in the sequestration of Undecaprenyl pyrophosphate. | bacitracin | 8 | 9 | 33 |
| 15 | *bcr* |  |  | 4 | 0 | 1 |
| 16 | *bl1_mox* | Class C beta-lactamase. This enzyme breaks the beta-lactam antibiotic ring open and deactivates the molecule's antibacterial properites. | cephalosporin | 1 | 0 | 0 |
| 17 | *bl2a_pc* | Class A beta-lactamase. This enzyme breaks the beta-lactam antibiotic ring open and deactivates the molecule's antibacterial properites. | penicillin | 2 | 2 | 0 |
| 18 | *bl2be_per* | Class A beta-lactamase. This enzyme breaks the beta-lactam antibiotic ring open and deactivates the molecule's antibacterial properites. | e_cephalosporin n_cephalosporin monobactam penicillin | 0 | 0 | 1 |
| 19 | *bl2c_bro* | Class A beta-lactamase. This enzyme breaks the beta-lactam antibiotic ring open and deactivates the molecule's antibacterial properites. | carbenicillin penicillin | 0 | 1 | 0 |
| 20 | *bl2c_pse1* | Class A beta-lactamase. This enzyme breaks the beta-lactam antibiotic ring open and deactivates the molecule's antibacterial properites. | carbenicillin penicillin | 0 | 0 | 2 |
| 21 | *ble* | Binding protein with a strong affinity to the bleomycin family of antibiotics, which confers resistance to these antibiotics by preventing the bleomycin-induced DNA breakage | bleomycin | 0 | 0 | 1 |
| 22 | *cata7* | Group A chloramphenicol acetyltransferase, which can inactivate chloramphenicol. | chloramphenicol | 3 | 2 | 1 |
| 23 | *cata8* | Group A chloramphenicol acetyltransferase, which can inactivate chloramphenicol. | chloramphenicol | 2 | 2 | 3 |
| 24 | *ceob* | Resistance-nodulation-cell division transporter system. Multidrug resistance efflux pump. | chloramphenicol | 0 | 0 | 3 |
| 25 | *cml_e3* | Major facilitator superfamily transporter, chloramphenicol efflux pump. | chloramphenicol | 0 | 1 | 1 |
| 26 | *cml_e8* | Major facilitator superfamily transporter, chloramphenicol efflux pump. | chloramphenicol | 0 | 0 | 2 |
| 27 | *dfra1* | Group A drug-insensitive dihydrofolate reductase, which can not be inhibited by trimethoprim. | trimethoprim | 1 | 0 | 2 |
| 28 | *emea* | Major facilitator superfamily transporter. Multidrug resistance efflux pump. | fluoroquinolone | 0 | 0 | 1 |
| 29 | *emrd* | Multidrug resistance efflux pump. |  | 0 | 0 | 1 |
| 30 | *ereb* | Erythromycin esterase, which can inactivate erythromycin by lactone ring cleavage. | erythromycin | 0 | 0 | 1 |
| 31 | *ermb* | rRNA adenine N-6-methyltransferase, which can methylate adenine at position 2058 of 23S rRNA, conferring resistance to erythromycin. | streptogramin_b lincosamide macrolide | 1 | 1 | 1 |
| 32 | *ermc* | rRNA adenine N-6-methyltransferase, which can methylate adenine at position 2058 of 23S rRNA, conferring resistance to erythromycin. | streptogramin_b lincosamide macrolide | 2 | 1 | 2 |
| 33 | *ermx* | rRNA adenine N-6-methyltransferase, which can methylate adenine at position 2058 of 23S rRNA, conferring resistance to erythromycin. | streptogramin_b lincosamide macrolide | 0 | 0 | 3 |
| 34 | *fosb* | Glutathione transferase, metalloglutathione transferase which confers resistance to fosfomycin by catalyzing the addition of glutathione to fosfomycin | fosfomycin | 5 | 0 | 5 |
| 35 | *ksga* | Specifically dimethylates two adjacent adenosines in the loop of a conserved hairpin near the 3'-end of 16S rRNA in the 30S particle. Its inactivation leads to kasugamycin resistance. | kasugamycin | 2 | 0 | 1 |
| 36 | *lnua* | Lincosamide nucleotidyltransferase. | lincomycin | 2 | 1 | 2 |
| 37 | *macb* | Resistance-nodulation-cell division transporter system. Multidrug resistance efflux pump. Macrolide-specific efflux system. | macrolide | 2 | 0 | 4 |
| 38 | *mdfa* |  |  | 0 | 0 | 1 |
| 39 | *mdth* | Major facilitator superfamily transporter. Multidrug resistance efflux pump. | deoxycholate fosfomycin | 0 | 0 | 1 |
| 40 | *mdtk* | Major facilitator superfamily transporter. Multidrug resistance efflux pump. | enoxacin norfloxacin | 1 | 0 | 4 |
| 41 | *mdtn* | Resistance-nodulation-cell division transporter system. Multidrug resistance efflux pump. | t_chloride acriflavine puromycin | 1 | 0 | 0 |
| 42 | *meca* | Penicillin binding protein, which has a low affinity for beta-lactams and catalyze a penicillin-insensitive transpeptidation. | beta_lactam | 0 | 0 | 7 |
| 43 | *mecr1* | Methicillin-resistance regulatory protein for mecA | methicillin | 0 | 1 | 2 |
| 44 | *mefa* | Major facilitator superfamily transporter, Macrolide-Lincosamide-Streptogramin B efflux pump. | macrolide | 1 | 0 | 0 |
| 45 | *mexb* | Resistance-nodulation-cell division transporter system. Multidrug resistance efflux pump. | tigecycline aminoglycoside fluoroquinolone beta_lactam tetracycline | 3 | 0 | 4 |
| 46 | *mexf* | Resistance-nodulation-cell division transporter system. Multidrug resistance efflux pump. | chloramphenicol fluoroquinolone | 2 | 1 | 0 |
| 47 | *mexw* | Resistance-nodulation-cell division transporter system. Multidrug resistance efflux pump. |  | 0 | 0 | 3 |
| 48 | *mexy* | Resistance-nodulation-cell division transporter system. Multidrug resistance efflux pump. | aminoglycoside glycylcycline | 0 | 0 | 2 |
| 49 | *mphc* | Macrolide phosphotransferase | macrolide | 3 | 2 | 4 |
| 50 | *msra* | ABC transporter system, Macrolide-Lincosamide-Streptogramin B efflux pump. | streptogramin_b lincosamide macrolide | 2 | 4 | 7 |
| 51 | *nora* |  |  | 4 | 4 | 9 |
| 52 | *pbp1a* | The enzyme has a penicillin-insensitive transglycosylase N-terminal domain (formation of linear glycan strands) and a penicillin-sensitive transpeptidase C-terminal domain (cross-linking of the peptide subunits) | penicillin | 0 | 0 | 1 |
| 53 | *pbp2* | The enzyme has a penicillin-insensitive transglycosylase N-terminal domain (formation of linear glycan strands) and a penicillin-sensitive transpeptidase C-terminal domain (cross-linking of the peptide subunits) | penicillin | 3 | 0 | 0 |
| 54 | *pbp2b* | The enzyme has a penicillin-insensitive transglycosylase N-terminal domain (formation of linear glycan strands) and a penicillin-sensitive transpeptidase C-terminal domain (cross-linking of the peptide subunits) | penicillin | 1 | 1 | 0 |
| 55 | *pbp2x* | The enzyme has a penicillin-insensitive transglycosylase N-terminal domain (formation of linear glycan strands) and a penicillin-sensitive transpeptidase C-terminal domain (cross-linking of the peptide subunits) | penicillin | 2 | 3 | 6 |
| 56 | *pmra* | Major facilitator superfamily transporter. Multidrug resistance efflux pump. | ciprofloxacin norfloxacin | 0 | 0 | 2 |
| 57 | *qac* | Small Multidrug Resistance (SMR) protein family. Multidrug resistance efflux pump, which consists of two proteins. | qa_compound | 0 | 0 | 2 |
| 58 | *qaca* | Multidrug efflux pump from bacterial pathogen staphylococcus aureus. Including QacA and QacB, both of which confer resistance to various toxic organic cations but differ in that QacB mediates lower levels of resistance to divalent cations. They differed by seven nucleotide subsititutions. | qa_compound | 0 | 0 | 1 |
| 59 | *qnrs* | Pentapeptide repeat family, which protects DNA gyrase from the inhibition of quinolones. | fluoroquinolone | 0 | 0 | 1 |
| 60 | *rosb* | Efflux pump/potassium antiporter system. RosA: Major facilitator superfamily transporter. RosB: Potassium antiporter. | fosmidomycin | 2 | 0 | 2 |
| 61 | *smee* | Resistance-nodulation-cell division transporter system. Multidrug resistance efflux pump. | fluoroquinolone | 0 | 0 | 1 |
| 62 | *str* | Streptomycin resistance protein. | streptomycin | 1 | 1 | 1 |
| 63 | *sul1* | Sulfonamide-resistant dihydropteroate synthase, which cannot be inhibited by sulfonamide. | sulfonamide | 0 | 0 | 4 |
| 64 | *sul2* | Sulfonamide-resistant dihydropteroate synthase, which cannot be inhibited by sulfonamide. | sulfonamide | 1 | 0 | 1 |
| 65 | *tet39* | Major facilitator superfamily transporter, tetracycline efflux pump. | tetracycline | 0 | 0 | 1 |
| 66 | *tetb* | Major facilitator superfamily transporter, tetracycline efflux pump. | tetracycline | 0 | 0 | 1 |
| 67 | *tetc* | Major facilitator superfamily transporter, tetracycline efflux pump. | tetracycline | 1 | 0 | 3 |
| 68 | *tetk* | Major facilitator superfamily transporter, tetracycline efflux pump. | tetracycline | 2 | 2 | 3 |
| 69 | *tetl* | Major facilitator superfamily transporter, tetracycline efflux pump. | tetracycline | 0 | 0 | 1 |
| 70 | *tetm* | Ribosomal protection protein, which protects ribosome from the translation inhibition of tetracycline. | tetracycline | 0 | 3 | 5 |
| 71 | *tetq* | Ribosomal protection protein, which protects ribosome from the translation inhibition of tetracycline. | tetracycline | 1 | 0 | 1 |
| 72 | *tett* | Ribosomal protection protein, which protects ribosome from the translation inhibition of tetracycline. | tetracycline | 0 | 0 | 1 |
| 73 | *tetw* | Ribosomal protection protein, which protects ribosome from the translation inhibition of tetracycline. | tetracycline | 0 | 0 | 1 |
| 74 | *tetx* | NADP-requiring oxidoreductase, an enzyme that can modify tetracycline. Mechanism detail unknown. | tetracycline | 0 | 0 | 1 |
| 75 | *tolc* | Resistance-nodulation-cell division transporter system. Multidrug resistance efflux pump. | aminoglycoside glycylcycline beta_lactam macrolide acriflavin | 1 | 1 | 2 |
| 76 | *vanya* | VanA type vancomycin resistance operon genes, which can synthesize peptidoglycan with modified C-terminal D-Ala-D-Ala to D-alanine--D-lactate. | vancomycin teicoplanin | 0 | 0 | 1 |
| 77 | *vgaa* | ABC transporter system, Macrolide-Lincosamide-Streptogramin B efflux pump. | streptogramin_a | 0 | 0 | 1 |
| 78 | *vanrc* | VanC type vancomycin resistance operon genes, which can synthesize peptidoglycan with modified C-terminal D-Ala-D-Ala to D-alanine--D-serine. | vancomycin | 2 | 0 | 0 |
